# Recent advances in managing chronic prostatitis/chronic pelvic pain syndrome

**DOI:** 10.12688/f1000research.10558.1

**Published:** 2017-09-25

**Authors:** Jaspreet Sandhu, Hin Yu Vincent TU

**Affiliations:** 1 Urology Service, Department of Surgery, Memorial Sloan Kettering Cancer Center, New York, USA

**Keywords:** prostatitis, chronic pelvic pain syndrome, phenotypically directed multimodal management, male pelvic pain, UPOINT

## Abstract

Chronic prostatitis/chronic pelvic pain syndrome is a common disorder seen in men under the age of 50 and has a considerable negative impact on quality of life; it is a complex and difficult condition to treat, owing to its wide symptomatology. In order to effectively treat this condition, the UPOINT system was developed: it allows clinical profiling of a patient’s symptoms into six broad categories (urinary symptoms, psychological dysfunction, organ-specific symptoms, infectious causes, neurologic dysfunction, and tenderness of the pelvic floor muscles) to allow individualized and multimodal therapy. In this review, we present the most recent advancements in the treatment of chronic prostatitis/chronic pelvic pain syndrome from the past few years.

## Introduction

Prostatitis is a common condition, with an estimated prevalence of 2 to 9% in the general male population ^1^. It has a significant negative impact on quality of life that is comparable to that of other chronic illnesses such as diabetes mellitus, Crohn’s disease, and myocardial infarction ^2^. With a wide range of symptomatology, patients with prostatitis can present with lower urinary tract symptoms (LUTS) and genital and pelvic pain ^3^. Perhaps much of the initial challenge in the management of these patients was understanding that the prostatitis “syndrome” consisted of multiple distinct clinical entities. In the validated classification system developed by the National Institutes of Health (NIH), prostatitis can be divided into four categories: acute bacterial prostatitis, chronic bacterial prostatitis, chronic prostatitis/chronic pelvic pain syndrome (CP/CPPS), and asymptomatic prostatitis ^4^. Accounting for more than 90% of urological outpatient cases seen, CP/CPPS remains one of the most common urologic disorders in men younger than 50 years old that is often poorly understood and complex to manage ^5^. Current multimodal treatments for CP/CPPS have been previously described in recent articles. After performing a literature search of PubMed, we present in this review the most recent advancements in the treatment of CP/CPPS over the last three years.

## UPOINT clinical phenotyping

The UPOINT system is a method to clinically profile a patient’s symptoms into six broad categories to allow individualized and multimodal therapy. Briefly, the six domains include urinary symptoms, psychological dysfunction, organ-specific symptoms, infectious causes, neurologic dysfunction, and tenderness of the pelvic floor muscles ^6^. Each domain is treated differently, allowing for a multimodal approach. Multiple clinical trials have externally validated the UPOINT system, and it is gaining increasing widespread use and effectiveness ^2^. Zhao *et al.* applied the system in a study of 389 patients in China and demonstrated that the number of positive UPOINT domains correlated well with a worse NIH chronic prostatitis symptom index (CPSI) total score ^7^. In a study of 100 patients utilizing UPOINT phenotyping, Shoskes *et al.* demonstrated that 84% of patients experienced a significant decrease in CPSI score at 26 weeks, defined as a six-point or greater decrease ^8^. Similarly, Magri *et al.* conducted a study of 914 patients using the UPOINT system and found that 77.5% of patients experienced a similar decrease in CPSI score that was durable at 18 months ^9^. Although further validation studies are required, such an approach provides physicians with a structured approach to an otherwise-challenging condition. For the purpose of this review, we present the latest novel treatments for the management of CP/CPPS based upon the UPOINT system.

## New therapies

### Urinary

There is significant heterogeneity in published data regarding the use of alpha-blockers and 5 alpha-reductase inhibitors (5-ARIs) as monotherapy. Smaller studies have suggested some clinical efficacy in the use of alpha-blockers for at least 12 weeks’ duration ^2^. In a randomized controlled trial, Nickel *et al.* reported that tamsulosin 0.4 mg daily significantly improved NIH-CPSI scores across multiple domains compared to placebo ^10^. In another study, silodosin 4 mg was shown to reduce NIH-CPSI scores compared to placebo ^11^. However, in a recent review published in *European Urology*, owing to the heterogeneity of the studies, the authors could not recommend alpha-blockers as first-line monotherapy, although it may be beneficial as part of multimodal therapy ^12^. Similarly, 5-ARIs could not be recommended as first-line hormonal therapy.

Studies have shown that phosphodiesterase-5 (PDE-5) inhibitors can improve LUTS by acting upon nitric oxide synthase and PDE-5 present within prostatic tissue. Park and colleagues reported that the use of mirodenafil, a newer PDE-5 inhibitor, in combination with levofloxacin versus levofloxacin alone improved the NIH-CPSI score by –7.2 and –3.2 ( *P* <0.05), respectively ^13^. Significant improvements were also noted in the International Prostatic Symptom Score (IPSS) voiding score and International Index of Erectile Function (IIEF) score.

### Psychosocial

Cannabis has been used in other chronic pain syndromes for symptom relief ^14^. Cannabidiol, a major component of cannabis, has been shown to suppress chronic inflammatory pain in mice by stimulating cannabinoid receptors ^15^. In a joint Canadian study with the Prostatitis Foundation, the authors surveyed men with CP/CPPS attending clinics and online on their experience with cannabis. A total of 342 participants responded to questions regarding their baseline NIH-CPSI scores, symptom improvement with cannabis usage, and side effects. The majority of patients reported that cannabis use made their mood, pain, and muscle spasms “slightly/much better”. The authors reported that 57% of clinic and 63% of online participants found cannabis was “somewhat” effective in treating their CP/CPPS symptoms ^14^. While cannabis may play a role in the complex management of CP/CPPS symptoms, it is important to note that multiple studies have shown an association between regular cannabis use and lower sperm concentration, lower total sperm count, and decreased sperm function ^16, 17^. As CP/CPPS is a disease common in men younger than the age of 50, the aspect of male fertility and desire for future offspring should be discussed prior to initiating therapy with cannabis ^9^.

### Organ specific

Phytotherapies, such as quercetin, saw palmetto, and cernilton, have been previously used with success to improve symptoms in patients with CP/CPPS ^18^. It has been hypothesized that this may be due to the antioxidant properties of phytotherapies, both as free radical scavengers and as xanthine oxidase inhibitors ^19, 20^. In a randomized trial, Iwamura *et al.* allocated 100 men to receive either Eviprostat or pollen extract ^21^. Response was defined as a decrease in the NIH-CPSI score by at least 25%. The authors reported that the response rate was 88.2% and 78.1%, respectively, with no significant difference between the two groups at 8 weeks. No adverse events were reported.

OM-89 is a novel oral immunostimulatory agent developed from lysed pathogenic *Escherichia coli* bacteria. Currently, other oral immunostimulatory agents are being used to treat inflammatory conditions such as rheumatoid arthritis ^22^. Given the success of these agents, Wagenlehner *et al.* enrolled 185 patients in a randomized double-blind placebo-controlled study to receive either OM-89 or placebo ^23^. The authors reported that although there was a mean decrease in the NIH-CPSI score of 40.5% and 44.0% in the treatment and placebo groups, respectively, and also a 67% and 64.3% positive response rate, respectively, their results did not reach statistical significance. Owing to the small sample size, no definitive conclusion could be drawn; however, OM-89 showed a positive response similar to placebo.

There is increasing evidence that lower-intensity pulsed ultrasound (LIPUS) can inhibit inflammation and pain by regulating the cyclooxygenase (COX-2) pathway ^24^. Li *et al.* randomized 96 CP/CPPS patients to treatment with transperineal ultrasound daily for 2 weeks versus sham treatment ^25^. They reported that both the treatment and the control arms had statistically significant improvement in pain, urinary symptoms, and quality of life scores. Between the two groups, statistically significant differences were observed in the total NIH-CPSI, pain, and urinary symptom scores after treatment with LIPUS.

### Infectious/inflammatory

Typically, patients presenting with CP/CPPS have already received multiple courses of antibiotics. However, in the absence of documented infection, the use of antibiotics in the treatment of CP/CPPS has been controversial ^2^. In a trial by Zhou *et al.*, 48 men with CP/CPPS were randomly assigned to treatment with tetracycline for 3 months versus placebo. They reported a significant decrease in the mean NIH-CPSI score by 18.5 points in the treatment group ^26^. Nonetheless, two other randomized controlled trials investigating the efficacy of ciprofloxacin or levofloxacin for a 6-week duration compared to placebo in the treatment of CP/CPPS did not reveal any significant difference in the change of NIH-CPSI score ^27, 28^. As such, there is currently not enough evidence to support the use of antibiotics in the primary treatment of CP/CPPS ^2, 12^.

Corticosteroids are not currently standard of care in the treatment of CP/CPPS; however, they may play a role in multimodal therapy. In a randomized double-blind parallel study, Yang *et al.* reported that men who received prednisone and levofloxacin for 2 weeks followed by levofloxacin for another 2 weeks had a statistically significant decrease in their NIH-CPSI, pain, and voiding score compared to the control group that received levofloxacin and placebo alone ^29^. They noted also a difference in the WBC count on extra-prostatic secretions at 4 weeks and no significant adverse effects. Newer second-generation formulations aimed at decreasing systemic activity while maintaining local efficacy such as beclomethasone dipropionate have also gained increasing interest. Bozzini *et al.* followed 180 men with CP/CPPS who received beclomethasone dipropionate suppositories and 136 of the 180 men who also received *Serenoa repens* extract ^30^. The authors noted that men in the steroid group versus steroid and *Serenoa repens* combined group had significant decreases in voiding frequency compared to baseline (–3.55 versus –3.68 voids per day, respectively) and increase in uroflowmetry (3.26 versus 5.61 mL/s, respectively). Both groups also had improvement in perineal pain on visual analog scale. The authors concluded that beclomethasone dipropionate suppositories were safe and observed improvement in LUTS and pain.

Although the incidence of fungal prostatitis is quite rare and beyond the scope of this article, studies have shown an increasing incidence of fungal UTIs, in part owing to the prevalent usage of broad-spectrum antibiotics ^31^. Kotb *et al.* investigated the efficacy of antifungals and urinary alkalinization with potassium citrate in 1,000 men who did not respond to 4 weeks of antibiotics and alpha-blockers ^31^. The predominant complaint was noted to be frequency and urgency, with vague genital discomfort. Of the 1,000 men, 803 reported at least 80% improvement in their urinary and pain symptoms, defined subjectively by the patient on a scale of 1 to 10. A lower initial prostate-specific antigen (PSA) and age were found to be associated with better response to antifungals.

### Neurologic/systemic

Neuromodulatory techniques have shown promising results in the treatment of CP/CPPS. In a randomized, placebo-controlled, double-blind trial, researchers from Switzerland investigated the effect of sono-electro-magnetic (SEM) therapy in the treatment of refractory CP/CPPS ^32^. The patients had tried other therapies including antibiotics, NSAIDS, and alpha-blockers for at least 6 weeks. Exclusion criteria included an NIH-CPSI score of less than 15, chronic bacterial prostatitis based on the Meares and Stamey glass test, post-void residual greater than 100 mL, prostate cancer, or urethral strictures. The device was applied over the perineum twice a day at home for 10 minutes using a portable device provided by the manufacturer. Both the active and the placebo devices functioned and appeared identical. At 12 weeks, there was no significant difference between the SEM and placebo group in NIH-CPSI score. However, subgroup analysis showed that the benefit with SEM was more pronounced than with placebo in patients with symptom duration of less than 12 months.

Although the exact mechanism of action is not fully understood, acupuncture has been used as a complementary therapeutic option in the management of refractory CP/CPPS in Eastern countries for some time ^33^. Its effects are hypothesized to be neuromodulatory in origin, but studies have shown that acupuncture may also increase endogenous opioid release and modulate sympathetic tone and pain pathways ^34, 35^. A randomized sham-controlled trial enrolled 100 patients to receive acupuncture at either seven acupoints bilaterally or sham points adjacent to these points ^35^. A baseline NIH-CPSI score was obtained and subsequently at 6, 8, 16, and 24 weeks after treatment. At 8 weeks, 48% of patients in the treatment group experienced complete resolution of symptoms as compared to 36% of sham participants. Although both groups experienced significant reduction in NIH-CPSI scores by the end of the 24 ^th^ week, the decline in scores remained significantly greater in the treatment group as compared to the sham group ( *P* <0.001). In a recent systematic review of acupuncture for CP/CPPS by Qin *et al.*, the authors concluded that, based on the current evidence, acupuncture is an effective treatment for CP/CPPS. When compared to sham acupuncture, real acupuncture was more effective at improving NIH-CPSI scores and subscores with a mean difference of –6.09 (95% CI: –8.12 to –5.68) ^36^. In contrast, when comparing acupuncture to medications, no definitive conclusions could be drawn owing to the heterogeneity and risk of bias of the included trials.

### Tenderness

There has been recent interest in targeting key modulators in the pain pathway and nerve growth factor (NGF) inhibition, all of which have been shown to reduce pain behaviors in animal models. Tanezumab, a monoclonal antibody against NGF, is such an agent. Nickel *et al.* performed a pooled analysis from three clinical trials of tanezumab in patients with chronic pelvic pain, including women with interstitial cystitis and men with CP/CPPS ^37^. In total, 208 patients were randomized to placebo or tanezumab, of which there were 38 and 41 men in the two groups, respectively. Pooled analysis of the three trials showed that although tanezumab provided some improvement in pain scores, these were not statistically significant in the male CP/CPPS population. The authors concluded that tanezumab remains a promising therapy requiring further studies, especially in the CP/CPPS population.

Botulinum neurotoxin-A (BoNT-A) is a toxin that is familiar to many urologists and is used in a variety of urologic conditions such as neurogenic detrusor overactivity. Studies have shown that its effects when injected intraprostatically include inhibiting the release of pain mediators and prostatic gland atrophy ^38^. In a study by Falahatkar *et al.*, 60 men with CP/CPPS underwent transurethral intraprostatic injection of BoNT-A or normal saline in a randomized double-blind study ^39^. Their results showed a statistically significant continuous and durable improvement in NIH-CPSI total and subscale scores at the final 6-month evaluation. Similar trends were observed in other instruments such as the AUA symptom score, visual analog scale, and quality of life scores. In contrast, in the placebo group, there was no significant change from baseline. No significant adverse events were recorded except for two patients who developed transient gross hematuria, which was managed conservatively. Given that the most prominent improvement was noted in the pain subscale, intraprostatic BoNT-A may be an option in patients with CP/CPPS and pain as a predominant symptom.

## Conclusion

CP/CPPS can be a challenging condition to treat, and a multimodal approach is usually required. Most of these patients have usually been on multiple courses of antibiotics without relief, leading to frustration among patients and practitioners. In addition to the current armamentarium of therapies, we present in this article a wide range of novel agents which have shown some clinical efficacy in the treatment of CP/CPPS. Many of these treatment strategies have shown promising results yet will require larger studies to demonstrate significant clinical impact.
